# In Vitro Characterization and Identification of Potential Probiotic Yeasts Isolated from Fermented Dairy and Non-Dairy Food Products

**DOI:** 10.3390/jof8050544

**Published:** 2022-05-23

**Authors:** Nadia S. Alkalbani, Tareq M. Osaili, Anas A. Al-Nabulsi, Reyad S. Obaid, Amin N. Olaimat, Shao-Quan Liu, Mutamed M. Ayyash

**Affiliations:** 1 Department of Food Science, College of Agriculture and Veterinary Medicine, United Arab Emirates University (UAEU), Al Ain P.O. Box 15551, United Arab Emirates; 950223010@uaeu.ac.ae; 2 Department Clinical Nutrition and Dietetics, University of Sharjah, Sharjah P.O. Box 27272, United Arab Emirates; tosaili@sharjah.ac.ae (T.M.O.); robaid@sharjah.ac.ae (R.S.O.); 3 Department of Nutrition and Food Technology, Jordan University of Science and Technology, Irbid 21121, Jordan; anas_nabulsi@just.edu.jo; 4 Department of Clinical Nutrition and Dietetics, Faculty of Applied Medical Sciences, The Hashemite University, P.O. Box 330127, Zarqa 13133, Jordan; aminolaimat@hu.edu.jo; 5 Department of Food Science and Technology, Faculty of Science, National University of Singapore, Singapore 117542, Singapore; fstlsq@nus.edu.sg

**Keywords:** autoaggregation, coaggregation, antimicrobial resistance, probiotics, yeast

## Abstract

This study is about the isolation of yeast from fermented dairy and non-dairy products as well as the characterization of their survival in in vitro digestion conditions and tolerance to bile salts. Promising strains were selected to further investigate their probiotic properties, including cell surface properties (autoaggregation, hydrophobicity and coaggregation), physiological properties (adhesion to the HT-29 cell line and cholesterol lowering), antimicrobial activities, bile salt hydrolysis, exopolysaccharide (EPS) producing capability, heat resistance and resistance to six antibiotics. The selected yeast isolates demonstrated remarkable survivability in an acidic environment. The reduction caused by in vitro digestion conditions ranged from 0.7 to 2.1 Log_10_. Bile salt tolerance increased with the extension in the incubation period, which ranged from 69.2% to 91.1% after 24 h. The ability of the 12 selected isolates to remove cholesterol varied from 41.6% to 96.5%, and all yeast strains exhibited a capability to hydrolyse screened bile salts. All the selected isolates exhibited heat resistance, hydrophobicity, strong coaggregation, autoaggregation after 24 h, robust antimicrobial activity and EPS production. The ability to adhere to the HT-29 cell line was within an average of 6.3 Log_10_ CFU/mL after 2 h. Based on ITS/5.8S ribosomal DNA sequencing, 12 yeast isolates were identified as 1 strain for each *Candida* *albicans* and *Saccharomyces cerevisiae* and 10 strains for *Pichia kudriavzevii*.

## 1. Introduction

Probiotics are defined as ‘live microorganisms that, when administered in adequate amounts, confer a health benefit on the host’ [1]. Probiotics contain various microorganisms, including bacteria and yeasts [2]. Lactic acid bacteria (LAB) and Bifidobacteria are the main sources of probiotic strains [3,4], which are widely used as supplements or in food industries. In contrast, to date, only a probiotic yeast, *Saccharomyces cerevisiae* var. *boulardii,* has gained the qualified presumption of safety (QPS) status from the European Food Safety Authority as a probiotic supplement [5]. *S. cerevisiae* var. *boulardii* is used in numerous countries to prevent and treat several gastrointestinal disorders [6]. However, the scientific community is witnessing a significant increase in the number of scientific studies on the isolation, characterization and identification of non-*Saccharomyces* yeasts (e.g., *Pichia*, *Schizosaccharomyces*, *Kluyveromyces*, *Rhodotorula* and *Candida*) and reporting them as promising probiotics [7,8,9,10].

Yeasts are unicellular eukaryotic microorganisms commonly found in soli, air, water, and food and are of animal and plant origin; they constitute <0.1% of microbiota in the human gut [11,12]. The use of yeasts as probiotics has gained increasing attention within the last few years, owing to their high contents of minerals, vitamin B, peptides, proteins and several immunostimulant compounds, such as mannan oligosaccharides, proteases and β-glucans [9,13,14]. Moreover, yeasts exhibit good resistance to industrial conditions, such as high temperature and lyophilization [15,16,17].

Currently, yeasts have gained increasing interest in the field of food biotechnology, including their roles in recombinant protein production, alcoholic fermentation and vitamin biosynthesis [9,18]. Furthermore, in the production of bread, beer, table olives, wine or kefir, yeasts are used as starters [19,20]. *Pichia* *kudriavzevii* and a combination of *S. cerevisiae* var. *boulardii* and inulin are used to produce fermented cereal-based food and symbiotic yogurt, respectively [21,22]. Yeasts are also associated with the maturation of certain cheeses [23]. Although yeasts may be a contaminant present in various foods (e.g., fruit juices, chocolate and yoghurt) that could cause food spoilage, many yeasts have been found to exhibit antimicrobial activity against foodborne pathogens and/or spoilage microorganisms [24,25].

The characterization of new probiotic candidates needs to follow the criteria established by the United Nations/World Health Organization (FAO/WHO) in 2002. The most important among these criteria is tolerance to the gastrointestinal tract (GIT) [26] conditions (low pH, digestive enzymes, bile salts and alkaline pH), adhesion to epithelial cells, bile salt hydrolysis (BSH), assimilation of cholesterol in the human intestine and food, antimicrobial activities and antibiotic sensitivity [1]. Furthermore, probiotic candidates should exhibit high-temperature tolerance for industrial purposes and the ability to produce exopolysaccharides (EPS) [27].

The biofunctional market continuously requires the diversification and application of novel products that provide new probiotic strains with specific functional properties [28]. Probiotic yeasts can provide functional properties that bacterial probiotics cannot. Thus, isolation of new probiotic yeasts is always required to meet the demands of the functional food and beverage market. The present study aimed (1) to isolate novel yeasts from dairy and non-dairy fermented food products, (2) to characterize the potential probiotic attributes of these newly isolated yeasts, including tolerance to the GIT conditions, cell surface and adhesive properties (autoaggregation, hydrophobicity, coaggregation and HT-29 cell line adhesion), antimicrobial activities, antibiotic sensitivities, heat tolerance, EPS production, ability to remove cholesterol and BSH activity, and (3) to identify the best potential probiotic yeasts using molecular techniques.

## 2. Materials and Methods

### 2.1. Sample Collection

A total of 105 samples of various fermented dairy and non-dairy food products sources free of any food preservatives were collected from different local markets in the United Arab Emirates (UAE). The samples were placed in an icebox and transported to the food microbiology lab of the UAEU for the isolation and characterization of the potential probiotic yeast strains. Unless otherwise stated, all chemicals were purchased from Sigma-Aldrich (St. Louis, MO, USA).

### 2.2. Isolation of Yeasts

The food samples were serially diluted with 1% peptone water (Neogen, Lansing, MI, USA). The pour-plate technique was employed using Yeast Extract–Peptone–Dextrose (YPD) agar (*Himedia* Laboratories Pvt. Ltd., Nashik, India), and the plates were aerobically incubated at 25 °C for 5 days (Binder C 170, Tuttlingen, Germany). Three copies of each colony isolates were subcultured in the YPD broth; subsequently, the stocks were prepared using glycerol (50% *v*/*v*) and then stored at −80 °C. The potential probiotic characteristics of the yeast isolates were evaluated after two successive activations at 25 °C.

### 2.3. Acid Tolerance: Preliminary Probiotic Investigation

Acid tolerance of the yeast isolates was evaluated at pH 2.5. A suspension of the tested yeast isolates was prepared in YPD broth and incubated at 25 °C for 24 h. The suspension was centrifuged at 5000× *g* for 10 min, washed with phosphate-buffered saline (PBS) (0.1 M, pH 7) and resuspended in 3 mL YPD broth with the pH adjusted to 2.5 using 1 M HCl. Subsequently, the suspension was distributed in 24-well plates and incubated at 25 °C for 24 h. A 1 mL solution of the resuspended yeasts pellets in a YEP broth without pH adjustment (pH 6.7) was considered a control. The growth levels of yeast strains were measured at OD_600_.

### 2.4. Tolerance to In Vitro Digestion Conditions

In vitro digestion tolerance was evaluated using the method described by Brodkorb et al. [29]. The in vitro gastrointestinal INFOGEST 2.0 protocol was applied to the yeast strains. A 2 mL aliquot of the yeast pellet suspension was subjected to in vitro digestion, including the oral (amylase 75 U/mL, salivary fluid SSF pH 7.0, 0.3 M CaCl_2_, 2 min, 37 °C), gastric (pepsin 2000 U/mL, RGE 60 U/mL, gastric juice SGF pH 3.0, 0.3 M CaCl_2_, 120 min, 37 °C) and intestinal (pancreatin 100 U/mL, bile 10 mmol/L, duodenal juice SIF pH 7.0, 0.3 M CaCl_2_, 120 min, 37 °C) phases. Continuous shaking at 120 rpm was applied during the in vitro digestion process. Serial dilution was performed to directly measure the yeast count before and after the in vitro digestion. 

### 2.5. Bile Salt Tolerance

The bile salt tolerance of the selected yeast isolates was tested according to AlKalbani et al. [30]. The selected yeasts were tested against 0.3% oxgall, 0.1% cholic acid and 0.1% taurocholic acid, individually, during 0, 6 and 24 h of incubation at 37 °C. The growth levels of yeast strains were recorded at OD_600_.

### 2.6. Cholesterol Removal

According to Alameri et al. [31], the capability of the selected yeast isolates to remove cholesterol was measured using *o*-phthalaldehyde at 550 nm. The cholesterol removal (%) was expressed as follows:

$$Cholesterol\;removal\;\left(\%\right)=\left[\frac{100-residual\;cholestrol\;at\;each\;incubation\;interval}{100}\right]\;\times \;\;100\;$$

### 2.7. Bile Salt Hydrolysis (BSH) Activity

The BSH activities were determined by measuring the amount of amino acids released from conjugated bile salts by yeast strains according to the method described by AlKalbani et al. [30]. The BSH activities were assayed against 6 mM sodium glycocholate, 6 mM sodium taurocholate or 6 mM conjugated bile salt mixture (glycocholic, glycochenodeoxycholic, taurocholic, taurochenodeoxycholic and taurodeoxycholic acids).

### 2.8. Autoaggregation

Autoaggregation assay of the activated cultures was performed according to the method described in [32], and absorbance was measured at 600 nm at the time intervals of 0, 3, 6 and 24 h. The autoaggregation percentage was calculated using the following equation:

(1) $$Auto-aggregation\left(\%\right)=\left[1-\frac{At}{A0}\right]\times \;100\;$$

where ‘*A_t_*’ denotes the absorbance at the time ‘*t*’, and ‘*A*_0_′ denotes the absorbance at the time ‘0′.

### 2.9. Hydrophobicity

Hydrophobicity was evaluated against three different hydrocarbons, n-hexadecane, xylene and octane, according to the method described by Fadda et al. [14]. The final absorbance was measured at 600 nm. The hydrophobicity percentage was expressed as follows:

$$Hydrophobicity\left(\%\right)=\left[\frac{A-A0}{A}\right]\times 100$$

where ‘*A*’ denotes the initial absorbance at 600 nm, and ‘*A*_0_′ denotes the final absorbance.

### 2.10. Coaggregation

The coaggregation experiment was conducted according to the method described by Andrade et al. [33] at 37 °C during incubation for 4, 6 and 24 h against four pathogens: *Escherichia coli* 0157:H7 1934, *Staphylococcus aureus* ATCC 25923, *Salmonella* Typhimurium 02–8423 and *Listeria monocytogenes* DSM 20649. The coaggregation percentage was calculated using the following equation:

$$Co-aggregation\left(\%\right)=\left[\frac{A0-At}{A0}\right]\times 100$$

where ‘*A_t_*’ denotes the absorbance at the time ‘*t*’, and ‘*A*_0_′ denotes the absorbance at the time ‘0′.

### 2.11. Antimicrobial Activity

The cell-free supernatant of the activated selected yeast isolates was used to determine the antibacterial activity against four foodborne pathogens: *L.* *monocytogenes*, *Salmonella* Typhimurium 02-8423, *E.* *coli* O157:H7 and *S.* *aureus*. The antimicrobial test was conducted according to the method described by Hossain et al. [34].

### 2.12. Antibiotic Susceptibility

The resistance of the selected yeast isolates to antibiotics (2-μg clindamycin (CLI), 10-μg ampicillin (AMP), 25-μg trimethoprim-sulfamethoxazole (SXT), 10-μg penicillin (PEN), 30-μg vancomycin (VA) and 15-μg erythromycin (E) (Oxoid; Hampshire, UK)) was evaluated using the YPD agar. This methodology was adapted from Tarique et al. [35]. The interpretative zones of resistant (R), moderately susceptible (MS) and susceptible (S) were defined according to the method described in [36].

### 2.13. Adhesion to the HT-29 Cell Line

To evaluate the adhesion ability of selected yeasts, the activated isolates were washed twice with Dulbecco’s phosphate-buffered saline. The adhesion property was tested according to the method described by Hong et al. [37] and measured in percentage using the following equation:

$$Adhesion\;ability\left(\%\right)=\left[\frac{At}{A0}\right]\times 100$$

where *A_t_* denotes the number of the adhered cells (log CFU/mL) after incubation, and *A*_0_ denotes the initial cell number (log CFU/mL).

### 2.14. EPS Production

The ability of the selected yeast isolates to produce EPS (−ve/+ve) was measured according to the method described by Angmo et al. [38], where yeasts cultured overnight were streaked onto the surface of plates containing ruthenium red milk agar (10% *w*/*v* skim milk powder, 1% *w*/*v* sucrose, 0.08-g/L ruthenium red, 1.5% *w*/*v* agar).

### 2.15. Heat Resistance

Heat resistance of the selected yeast isolates was measured according to the method described by Teles Santos et al. [39] at 60 °C for 5 min. Serial dilution was performed to directly measure the yeast count before and after heat treatment. 

### 2.16. Molecular Identification of the Selected Yeast Isolates

A total of 12 yeasts were selected and subjected to PCR amplification of the ITS/5.8S ribosomal DNA. DNA extraction and purification were performed using DNeasy UltraClean Microbial Kit (Qiagen, Carlsbad, CA, USA) and PCR Kit (BIONEER, Daejeon, Korea) according to the manufacturer’s protocols. PCR analysis was conducted as detailed in [40,41] and according to Amorim et al. [7] using primers ITS1 (5′-TCCGTAGGTGAACCTGCGG-3′) and ITS4 (5′-TCCTCCGCTTATTGATATGC-3′). Sequencing was performed at the Macrogen sequencing facilities (Macrogen-Korea, Seoul, Korea). Yeast identification was achieved by comparing the obtained sequences with those available from the NCBI database using the BLAST algorithm. The accession numbers of the selected yeast isolates were obtained by GenBank^®^. The neighbour-joining method was employed to determine the closest yeast species using the MEGA software version 11 [42,43].

### 2.17. Statistical Analysis

To determine whether the variations between yeast isolates had a significant influence on quantitative parameters, one-way ANOVA and Tukey’s test were conducted to examine the differences between the mean values at *p* *<* 0.05. All tests were conducted at least three times.

## 3. Results and Discussion

A total of 105 colonies with different morphological properties were isolated on YPD agar from different food products sold in the local market. The selected yeast isolates were purified and preserved at −80 °C in 50% glycerol containing YPD broth.

### 3.1. Preliminary Acid Tolerance

**Figure 1:**
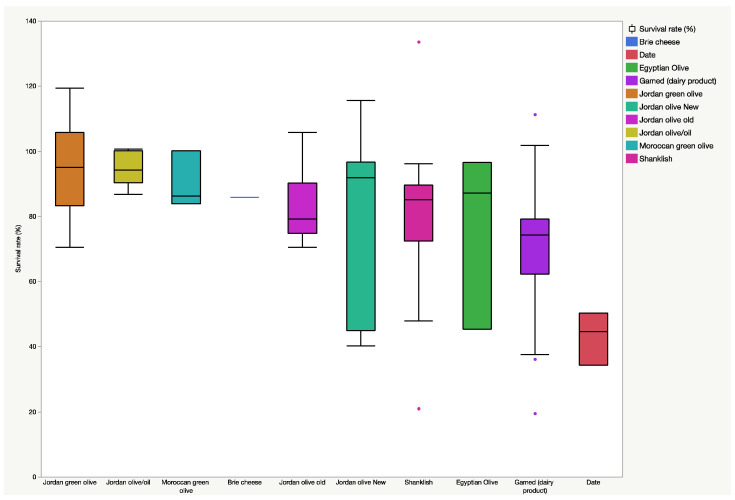
Boxplot summarizing the survival rate (%) of the 105 yeast isolates under pH 2.5 for 2 h at 37 °C. Bullets represent outliners.

The acid tolerance percentages of 105 isolates at pH 2.5 during 24 h of incubation at 37 °C are presented in Table S1 and summarized in Figure 1 (boxplot). The yeasts isolates exhibited various levels of survivability at low pH (0.0% to 100%). A total of 45 yeast isolates that demonstrated noticeable acid tolerance were selected to investigate their tolerance to in vitro digestion conditions and bile salt.

The beneficial aspects of probiotics can be exploited if they exhibit resistance to an acidic environment. Thus, acid tolerance is a pivotal factor that allows the candidate probiotic to pass through the gastrointestinal tract (GIT) in a vital and adequate amount and to be used in the food industry. In this study, a low acidic medium pH of 2.5 at 37 °C was used as a preliminary indicator for potential probiotic features that could be held in our isolates. Generally, adjustment of yeast cell walls and activation of the cell wall integrity and general stress response pathways are the main strategies that enable the selected probiotic yeasts to resist a strong inorganic acid [44,45].

In the present study, high survivability in an acidic medium is preferred. The strains were basically isolated from low-pH environments such as fermented dairy and non-dairy products, where they cohabited with the lactic and/or acetic acid produced by bacteria. In this context, the results of Santos et al. [46] and Moreira et al. [47] are consistent with ours. Şanlidere Aloğlu et al. [48] tested the different yeast species they collected at pH 2.5 according to our acid tolerance conditions.

### 3.2. Tolerance to In Vitro Digestion Conditions and Bile Salts

Table 1 presents the survival rates of potential yeast probiotics before and after being subjected to in vitro digestion with simulated fluids and bile stress against oxgall, cholic acid and taurocholic acid at different concentrations. The growth of all yeast isolates decreased (*p* < 0.05) under in vitro digestion conditions. The yeasts’ count reduction after in vitro digestion ranged from ~0.7 to 2.1 Logs. In general, isolates O63, SH45, SH40, O12, O26, SH46 and SH55 exhibited the highest resistance to in vitro digestion conditions. On the other hand, the yeast isolates demonstrated remarkable resistance to oxgall compared with cholic and taurocholic acids. The bile salt tolerance of the yeast isolates increased with the extension in the incubation period, which ranged from 43.8% to 87.9%, 17.4% to 85.7% and 68.4% to 86.7% after 6 h and from 48.9% to 90.5%, 26.5% to 89.5% and 69.2% to 91.1% after 24 h. Overall, isolates SH104, SH105, SH 96, G1, SH46, O12 and O24, among others, exhibited high bile resistance. Twelve isolates with high survivability in in vitro digestion conditions were selected according to their varying isolated sources for subsequent investigations. These isolates were G1, O12, O13, O18, O21, O26, O36, O63, O66, SH40, SH45 and SH55.

A probiotic candidate must exhibit high survivability in stressful conditions that it will inevitably face inside the human gastrointestinal tract (GIT) to exert its functionality. At the start of the digestion process, the potential probiotics should demonstrate tolerance to the amylase present in the oral cavity. After ingestion, the potential probiotics must resist several harsh conditions in the stomach, e.g., presence of low pH, gastric fluid and pepsin [49]. Next, the probiotic cells must exhibit resistance to the small intestine conditions, such as the presence of pancreatin, bile salts and alkaline stress [28]. Moreover, tolerance to mild heat shock is necessary for the survivability of probiotic strains. The probiotic candidate has to retain its viability and functionality at the internal temperature of the human body (37 °C) because 28–30 °C is mostly the optimal temperature for yeast growth [50].

Consequently, the potential probiotic should exhibit low reduction in viability after being subjected to in vitro digestion [51]. Generally, the yeast probiotic tolerance mechanism to the GIT conditions depends on the species/strain. Bile salts possess antimicrobial activity that could suppress any microorganism, including yeasts. Thus, for microorganisms to be classified as probiotics, they need to resist bile salts. The bile salt resistance of *S. cerevisiae* could be attributed to an increase in its lipid content after being exposed to bile salts and low pH. These lipids contents probably act as a protective agent against bile salt stress [52,53].

In light of our results, the resistances of all isolates to the GIT conditions and bile salts are remarkably different depending on the species/strain specificity. Other works yielded promising findings for *P. kudriavzevii* [54] and *S. boulardii* var*. boulardii* strains [55], which tolerated simulated GIT juices, isolated from fermented cereal foods and commercial food supplements. In agreement with our findings, Chen et al. [56], Menezes et al. [57] and Amorim et al. [7] proved the capability of different yeast strains isolated from a variety of food sources to tolerate bile salt.

### 3.3. Cholesterol Removal and Bile Salt Hydrolysis (BSH)

Table 2 presents the cholesterol removal and BSH activities of 12 yeast strains. All 12 yeast strains were capable of effectively removing cholesterol from YPD media. Table 2 demonstrates that the cholesterol removal ability significantly differed among the yeast strains, which varied from 41.6% to 96.5%. Strains O21, O26, SH55 and O13 exhibited a higher ability to remove cholesterol compared with the other investigated yeast strains. Regarding BSH, all yeast strains exhibited the capability to hydrolyse screened bile salts forming free cholic acid. This capability ranged from 3.48 to 4.62, 3.40 to 4.01 and 3.56 to 4.77 U/mg for sodium glycocholate, sodium taurocholate and mixture of bile salts, respectively. Strains O12, O26 and O66 demonstrated higher BSH activities than the other investigated yeast strains (Table 2).

Cholesterol removal is one of the desirable features of probiotics. In the current study, the investigated isolates exhibited cholesterol reduction capability and BSH activities. Cholesterol assimilation by a probiotic microorganism has been attributed to four main mechanisms, namely, attachment to the cell wall, reduction of cholesterol to coprostanol, incorporation of the cholesterol in the cell wall and disruption of the cholesterol micelles by BSH [58,59]. Our findings on the cholesterol-lowering ability of the isolated yeasts are superior to those reported in [48,60,61,62].

Probiotics possess BSH activities to act as bile salt detoxifiers and promote competition in the microbial communities within the small intestine [63,64]. The ability of probiotic strains to resist the toxicity of conjugated bile salts present in the duodenum is associated with their BSH activity. In agreement with our results, Fadda et al. [14] and Şanlidere Aloğlu et al. [48] reported several yeast strains isolated from foods exhibiting BSH activity.

### 3.4. Autoaggregation and Hydrophobicity

Table 3 presents the autoaggregation (%) during 24 h of incubation at 37 °C and hydrophobicity (%) against hexadecane, xylene and octane. The 12 yeast isolates exhibited a significant percentage of autoaggregation ranging from 37.6% to 66%, 44.5% to 84.0% and 50.7% to 85.8% during 3, 6 and 24 h of incubation, respectively. In general, the autoaggregation percentages increased with the increase in the incubation period. After 24 h, isolates SH45, O36, O26, O66, O23, O28 and O21 showed a higher autoaggregation ability than the other screened isolates. Table 3 demonstrates that the hydrophobicity of the 12 isolates to hexadecane and octane was higher than to xylene. The hydrophobicity percentages ranged from 23% to 50.4%, 28.2% to 46.5% and 4.3% to 42.5% for hexane, octane and xylene, respectively (Table 3). Isolates SH40, O36, O40, O36, O12, O21 and O26 presented higher hydrophobicity than the other evaluated isolates.

The adherence of microorganisms to epithelial cells in the human intestine can be deduced by their cell surface properties, represented by testing the autoaggregation capability and hydrophobic properties of probiotic candidates [65]. A higher aggregation capacity provides high cell intensity involving the adhesion mechanism, whereas a robust hydrophobic property facilitates the attachment between the microbe and epithelial cells [28]. In the present study, the yeast strains exhibited significant percentages of autoaggregation and hydrophobicity to the investigated hydrocarbons. However, there were remarkable distinctions among the screened isolates, which may be attributed to the difference in the hydrophilic and hydrophobic regions in the cell wall of the microbial isolates [66]. In addition, Verstrepen and Klis [67] reported that the differential expression of the adhesin genes in the yeast allows them to rapidly adjust their adhesive properties to a specific environment. It is noteworthy that the size of the yeasts cell are 10 times larger than that of bacteria [12]. Therefore, an individual yeast cell requires a larger area to adhere to the human intestinal cell surface [68]. 

In this work, the increasing trend of autoaggregation throughout 24 h is consistent with the findings of Bonatsou et al. [32], whereas both the autoaggregation and hydrophobicity results are superior to those reported by Zullo and Ciafardini [62]. The drawback of the latter study [62] was that the hydrophobicity of yeasts was examined against one hydrocarbon (hexadecane). Moreover, the autoaggregation capacity of the yeasts was tested for only 4 h.

### 3.5. Coaggregation and Antimicrobial Activity

The coaggregation percentages of 12 yeast strains in the presence of *E. coli O157:H7*, Salmonella Typhimurium, L. monocytogenes and *S. aureus* at 3, 6 and 24 h of incubation at 37 °C and antimicrobial activities against the same four pathogens are presented in Table 4. The coaggregation capability increased (*p* < 0.05) during the incubation period of 3 to 24 h at 37 °C, particularly with *Salmonella Typhimurium*. However, from another view, the yeast isolates had the highest coaggregation percentages with *L. monocytogenes* than the other three pathogens during the incubation period. Overall, isolates O12, O21, O26, O66 and SH45 had a higher coaggregation percentage than the other investigated strains. The antimicrobial activity presented in Table 4 ranges from 0.1 to >2.0 mm zone. Interestingly, all yeast strains exhibited substantial inhibition activities against all four pathogens, except the G1, O26 and O13 isolates.

The capability of the probiotics to coaggregate with the foodborne pathogens and their potential to displace these pathogens are critical for protection against enteric infections [69]. Yeast probiotics prevent the pathogens from adhering to the intestinal epithelial cells by adhering to them instead and then cocurating their binding sites [33]. Generally, probiotics adapt a coaggregation behaviour to form a competitive microenvironment surrounding the pathogen [70]. The suggested coaggregation mechanism between yeasts and bacterial pathogens has been proposed by Millsap et al. [71], who stated that particular bacterial pathogens have binding molecules on their surfaces that allow them to bind to mannose residues on the yeast cell surface. In addition to mannans, glucans and chitin, which are the main components of the yeast cell wall, all may be associated with yeast coaggregation with pathogenic bacteria [18]. Several studies have also confirmed particular pathogenic bacteria bound to *S. boulardii*, *Debaryomyces hansenii* and *Yarrowia* *lipolytica* [72,73,74]. Our strains exhibited an intermediate coaggregation ability. However, the higher coaggregation results for all four investigated pathogens are superior to those for *Kluyveromyces lactis* and *Torulaspora delbrueckii* toward the same four pathogens [33].

The antimicrobial activity of probiotics is an essential characteristic represented by antimicrobial compound production, completing exclusion of the pathogens and promotion of the intestinal barrier function [75]. Several mechanisms have been postulated for antagonistic yeasts against pathogenic bacteria, including (1) competition for nutrients and space between yeast probiotic and microbial pathogens; (2) pH changes in the environment due to the metabolic activity of the yeasts, leading to stressful conditions for the pathogens; (3) production of high-concentration ethanol; and (4) release of antibacterial substances and secretion of antimicrobial compounds, such as mycocins or killer toxins [18,76,77,78]. In this work, *P.* *kudriavzevii* represents the majority of the tested isolates, and it belongs to the *Pichia* genus, which was deeply reviewed as a producer of killer toxins that can inhibit particular pathogens by Belda et al. [79].

Our antimicrobial activity results are in contrast to those of Amorim et al. [7] because no antimicrobial activity was exhibited by their tested yeast isolates (*Candida lusitaniae* and *Meyerozyma caribbica*). However, the results obtained by Hossain et al. [34] coincide with the current study. Furthermore, the results of the current study indicated that the differences in the antimicrobial activity among the yeast isolates might be attributed to species and strain specificity.

### 3.6. Antibiotic Susceptibility and Attachment to the HT-29 Cell Line

The antibiotic resistance of 12 yeast strains against 6 antibiotics is presented in Table 5. All yeast strains were sensitive or moderately sensitive to all the investigated antibiotics, except strains G1, O12, O13 and O26. Table 5 demonstrates that the yeast strains were more susceptible to erythromycin and clindamycin. Regarding the HT-29 cell line adhesion, the range of the yeasts’ adhesion to the HT-29 cell line was 5.97–6.99 Log_10_ CFU/mL (Table 5). Generally, isolates G1, O12, O13 and SH45 had the highest ability for HT-29 cell line attachment.

The antibiotic resistance of probiotics is deemed a safety concern because there is a chance of an antimicrobial resistance gene horizontally transmitting to the pathogens [28]. Therefore, potential probiotics with antibiotic sensitivity are desirable. In our work, eight strains were found to be susceptible or moderately susceptible to various commercial antibiotics. Our results are almost in line with those of Amorim et al. [7] and Hossain et al. [34], who isolated yeast species from pineapple and soya paste, respectively. The minor disparities between our study and others can be attributed to strain and species variations.

The capability to adhere to the intestinal epithelium is one of the primary criteria for probiotic candidate selection. This capability is considered a pre-condition to exclude enteropathogenic bacteria or promote host immunomodulation [80,81]. Expressed proteins located on the surface of the cell walls are associated with microbial adhesion to intestinal epithelial cells [68,82]. Generally, the results obtained from the present work showed suitable attachment to the HT-29 cell line. Several studies verified the adhesion abilities of different yeast strains isolated from food sources using the HT-29 cell line [37,60,83].

### 3.7. EPS Production and Heat Resistance

Interestingly, all 12 isolates showed the potential to produce EPS, as presented in Table 6.

The EPS production of the yeast isolates was inferred by creating a white ropy mucus on ruthenium red skim milk agar plates. Numerous microorganisms, including yeasts, can produce EPSs, which may vary in their monomer composition, molecular weight and type and degree of branching [84]. Therefore, EPSs differ in their functions and applications, which are most related to adhering to, protecting and retaining compounds [85]. The research group [86] had reported EPS production and isolation by yeast, *K. marxianus* and P. kudriavzevii, which were isolated from dairy products. On the other hand, Fekri et al. [87] revealed that their p yeast strains isolated from traditional sourdough, *K.* *marxianus*, *K.* *lactis* and *K.* *aestuarii*, produced a higher amount of EPS compared with those of isolated yeasts in the same research [87].

The heat resistance of 12 yeast isolates is presented in Table 6. The growth of all isolates reduced (*p* < 0.05) after they were treated at 60 °C for 5 min. The decrease in yeast growth ranged from 1.7 to 2.6 Log_10_ CFU/mL. Isolates O18, O21, O63 and SH40 presented higher heat resistance compared with other isolates.

Heat resistance is a fundamental challenge faced by probiotics when used in the food industry. In the present study, all yeast isolates demonstrated good tolerance to heat. One of the suggested mechanisms for the yeasts to resist extreme heat is the production of trehalose, a sugar produced by a wide variety of microorganisms. The intracellular accumulated trehalose is involved in promoting thermotolerance of the yeasts [88]. Several studies have evaluated the heat resistance of yeast probiotics using a method that mainly focuses on testing at only 37 °C, which is the internal temperature of the human body [9,89,90]. The drawback of this method is that it only evaluates the use of probiotics as a supplement, not its use in the food industry, which requires higher temperature. In the studies conducted by Hu et al. [91] and Hossain et al. [34], the heat resistance of *S. cerevisiae* and *S.* *cerevisiae* var*. boulardii* was tested up to 42 °C and 48 °C for 30 min and 72 h, respectively. The isolates in both studies [34,91] exhibited a significant reduction in growth rate after heat treatment compared with our isolates. The trend of the heat resistance of *S. cerevisiae* has been reported by Kalyuzhin [92].

### 3.8. Molecular Identification of Selected Yeast Isolates

**Figure 2:**
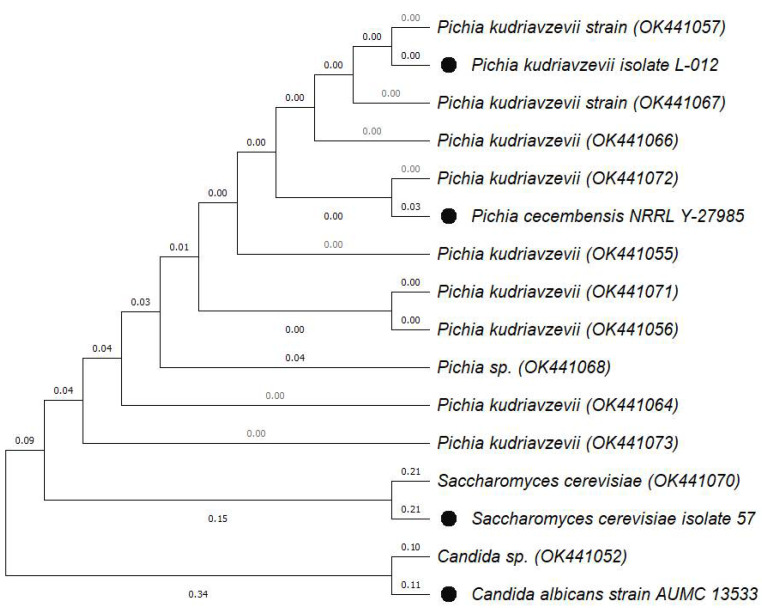
Neighbour-joining phylogenetic tree based on ITS/5.8S ribosomal DNA. The numbers in parentheses are accession numbers of the identified sequences from the GenBank. The filled circles are the reference strains from NCBI.

A total of 12 potential yeast probiotics were identified using ITS/5.8S ribosomal DNA sequences. Each isolate’s name and accession number obtained from GenBank are presented in Table 7. Molecular phylogeny analysis was conducted, and a phylogenetic tree constructed to identify yeasts to a species level based on the 1ITS/5.8S ribosomal DNA sequences from evolutionary distances using the neighbour-joining method. The phylogenetic tree of the 12 isolates is presented in Figure 2. The genotyping of *S. cerevisiae,* one of the yeast species included in the current paper, has been widely discussed [93,94]. One of the most reliable methods used to amplify the genomic sequences is PCR amplification of inter-delta sequences, where delta elements create the LTR flanking retrotransposons TY1 and TY2 in *S. cerevisiae* [41]. Therefore, in order to distinguish the *S. cerevisiae* strain, the use of inter-delta sequencing is recommended.

## 4. Conclusions

Selected yeast strains from fermented dairy and non-dairy products demonstrated probiotic characteristics. The probiotic yeasts exhibited an excellent survival rate after the in vitro digestion, with a 0.7 Log reduction for the highest in vitro digestion resistance. The yeast isolates were able to hydrolyse bile salts and significantly reduce cholesterol. The susceptibility of these strains to the tested antibiotics did not present any concerns. The autoaggregation of 12 isolates ranged from 50.7% to 85.8% during 24 h of incubation. All those isolates exhibited a higher percentage of hydrophobicity to hexadecane and octane compared with xylene. Generally, the increase in coaggregation percentages during incubation time from 3 h to 24 h was remarkable (*p* < 0.05). The isolates showed significant inhibition activities against the four screened pathogens except G1, O26, and O13 isolates. Overall, the 12 isolates had moderate ability to attach to the HT-29 cell line. The reduction in the growth of 12 isolates after heat treatment ranged from 1.7 to 2.6 LoG_10_ CFU/mL. All the yeast isolates can produce exopolysaccharides (EPS), and isolates SH40 (*Pichia kudriavzevii* OK441071), SH55 (*P. kudriavzevii* OK441073), O63 (*Picha* sp. OK441068) and O66 (*S. cerevisiae* OK441070) have promising probiotic traits, which necessitate further characterization for their use in the food industry.

## Figures and Tables

**Table 1:** In vitro digestion conditions and bile salt tolerances for 45 potential probiotic yeast isolates.

Isolate	Tolerance to GIT			Bile Salt Tolerances (%)					
				6 h			24 h		
	Before	After	Log Reduction	0.3 CA	1.0 TA	1.0 OX	0.3 CA	1.0 TA	1.0 OX
G.1	7.3 ± 0.01	5.5 ± 0.03	1.8	54.2	36.9	74.1	68.3	81.4	89.1
G.2	7.5 ± 0.09	5.4 ± 0.02	2.1	53.7	36.1	74.8	66.7	73.5	88.1
G.3	7.6 ± 0.24	6.2 ± 0.12	1.4	71.6	52.5	70.7	84.3	82.1	83.3
G.6	7.4 ± 0.09	6.3 ± 0.11	1.1	73.8	61.6	77.2	78.9	81.1	87.5
G.7	7.4 ± 0.13	6.1 ± 0.12	1.3	66.4	68.6	79.7	80.8	83.4	88.0
G.8	7.5 ± 0.11	6.1 ± 0.06	1.4	71.0	81.7	79.6	83.8	85.6	88.3
G.9	7.5 ± 0.07	6.2 ± 0.10	1.3	67.8	76.8	80.0	79.9	81.5	84.5
G.10	7.5 ± 0.02	6.2 ± 0.02	1.4	70.5	64.7	80.8	83.0	80.7	87.6
O.12	7.3 ± 0.06	6.3 ± 0.04	0.9	80.5	78.9	81.0	87.4	84.1	87.4
O.13	7.5 ± 0.06	6.2 ± 0.03	1.3	72.8	68.2	79.2	80.5	81.1	87.1
O.18	7.5 ± 0.01	6.3 ± 0.02	1.2	67.0	80.0	84.8	79.9	81.4	88.7
O.19	7.4 ± 0.04	6.1 ± 0.03	1.3	69.0	45.9	85.8	78.2	58.0	86.2
O.20	7.5 ± 0.02	6.3 ± 0.01	1.2	79.8	42.4	80.6	86.1	81.5	87.1
O.21	7.6 ± 0.19	6.4 ± 0.20	1.3	82.2	59.2	86.3	86.9	80.7	89.1
O.22	7.5 ± 0.04	6.3 ± 0.05	1.2	82.9	76.7	84.2	87.5	79.4	87.9
O.23	7.4 ± 0.08	6.2 ± 0.03	1.2	73.7	67.8	83.8	79.8	75.6	88.0
O.24	7.5 ± 0.03	6.5 ± 0.09	1.0	84.5	70.3	86.7	87.9	83.7	91.1
O.26	7.4 ± 0.06	6.2 ± 0.09	1.2	80.3	69.5	82.0	83.4	77.9	88.2
O.30	7.5 ± 0.01	6.3 ± 0.10	1.2	67.0	61.6	79.7	78.7	75.4	86.0
O.33	7.4 ± 0.03	6.4 ± 0.06	0.9	73.9	62.7	80.5	83.1	69.9	83.9
O.36	7.4 ± 0.05	6.2 ± 0.01	1.3	84.3	79.2	84.3	87.7	85.7	89.8
SH.40	7.4 ± 0.08	6.6 ± 0.09	0.9	73.7	63.4	81.3	81.4	77.9	86.9
SH.45	7.1 ± 0.02	6.1 ± 0.04	1.0	65.1	62.7	84.0	84.6	74.4	86.1
SH.46	7.2 ± 0.10	6.3 ± 0.11	0.9	70.5	63.4	82.2	85.0	76.6	90.7
SH.55	7.0 ± 0.24	6.0 ± 0.16	1.0	73.2	68.2	72.7	86.0	80.9	84.4
O.63	7.1 ± 0.12	6.4 ± 0.08	0.7	64.2	64.8	81.4	66.9	65.9	86.4
O.65	7.2 ± 0.00	6.3 ± 0.04	1.0	68.0	62.7	81.4	77.1	75.1	86.7
G.69	7.3 ± 0.06	6.3 ± 0.06	1.0	43.8	17.4	68.6	48.9	26.5	69.8
O.66	7.2 ± 0.15	5.4 ± 0.07	1.8	53.1	51.7	69.2	57.5	66.0	69.9
G.75	7.4 ± 0.20	5.9 ± 0.11	1.5	52.5	57.0	69.4	57.5	61.3	70.3
G.71	7.5 ± 0.13	6.2 ± 0.16	1.3	76.4	70.4	72.7	86.6	82.7	83.3
G.77	7.2 ± 0.17	5.8 ± 0.12	1.4	78.5	75.7	76.6	87.2	87.3	85.6
G.78	7.1 ± 0.06	6.1 ± 0.04	1.0	81.7	72.7	77.3	88.1	85.5	86.1
G.80	7.2 ± 0.21	5.9 ± 0.10	1.3	67.2	78.0	76.5	80.3	84.3	86.4
G.82	7.4 ± 0.09	5.9 ± 0.06	1.5	78.1	70.1	75.5	85.9	81.1	84.8
G.84	7.2 ± 0.08	5.9 ± 0.04	1.3	78.9	70.7	72.0	87.8	80.8	86.0
SH.96	7.4 ± 0.17	5.8 ± 0.14	1.6	86.8	84.8	75.7	89.7	89.2	90.1
SH.97	7.4 ± 0.08	6.2 ± 0.04	1.3	85.9	84.8	78.1	90.2	89.3	89.8
SH.98	7.2 ± 0.23	5.7 ± 0.09	1.5	87.3	82.4	71.9	89.5	88.8	81.9
SH.99	7.3 ± 0.32	6.3 ± 0.27	1.0	84.9	77.3	68.4	89.6	89.0	69.2
SH.100	7.4 ± 0.13	5.9 ± 0.18	1.5	84.9	83.0	81.1	89.8	89.1	90.3
SH.102	7.3 ± 0.13	5.6 ± 0.07	1.7	83.9	85.7	73.3	89.4	88.7	89.4
SH.103	7.1 ± 0.19	5.5 ± 0.17	1.6	87.9	81.7	79.9	90.8	89.5	90.3
SH.104	7.4 ± 0.30	6.2 ± 0.22	1.2	86.6	79.8	80.0	90.5	89.4	90.6
SH.105	7.4 ± 0.14	6.1 ± 0.08	1.3	86.3	70.5	74.8	89.4	87.8	89.4

Values are expressed as mean ± standard deviation of triplicates. CA, cholic acid; OX, oxgall; TA, taurocholic acid. GIT, stimulated gastrointestinal tract by INFOGEST.

**Table 2:** Cholesterol removal (%) and bile salt hydrolase (BSH) activities (specific activity, U/mg) of 12 potential probiotic yeasts.

Isolate	CR (%)	BSH					
		Na-SG	SA	Na-TA	SA	Bile salt mixture	SA
G1	47.98 ± 7.55 ^ab^	1.79 ± 0.05 ^abc^	3.70	1.83 ± 0.07 ^bc^	3.79	1.72 ± 0.05 ^a^	3.56
O12	50.16 ± 8.68 ^ab^	1.80 ± 0.07 ^bc^	3.68	1.72 ± 0.07 ^ab^	3.52	1.84 ± 0.07 ^bc^	3.77
O13	71.96 ± 5.20 ^d^	2.13 ± 0.10 ^e^	4.46	1.88 ± 0.04 ^c^	3.93	1.73 ± 0.07 ^a^	3.62
O18	62.31 ± 2.35 ^cd^	1.90 ± 0.06 ^d^	3.85	1.72 ± 0.04 ^ab^	3.49	2.11 ± 0.08 ^d^	4.27
O21	95.02 ± 1.43 ^e^	1.87 ± 0.03 ^cd^	4.01	1.70 ± 0.06 ^a^	3.65	2.22 ± 0.05 ^e^	4.77
O26	91.59 ± 2.47 ^e^	2.17 ± 0.03 ^ef^	4.55	1.91 ± 0.02 ^c^	4.01	2.26 ± 0.04 ^e^	4.73
O36	53.58 ± 1.08 ^bc^	1.89 ± 0.02 ^d^	3.95	1.82 ± 0.05 ^abc^	3.81	1.92 ± 0.05 ^c^	4.02
O63	47.98 ± 1.95 ^ab^	1.74 ± 0.04 ^ab^	3.57	1.71 ± 0.05 ^ab^	3.50	1.89 ± 0.04 ^bc^	3.87
O66	65.42 ± 2.80 ^cd^	1.94 ± 0.04 ^d^	4.04	1.88 ± 0.02 ^c^	3.90	2.04 ± 0.05 ^d^	4.25
SH40	39.56 ± 2.86 ^a^	1.76 ± 0.02 ^ab^	3.48	1.71 ± 0.02 ^ab^	3.40	1.81 ± 0.02 ^ab^	3.59
SH45	59.81 ± 1.87 ^bc^	1.71 ± 0.05 ^a^	3.48	1.71 ± 0.02 ^ab^	3.48	1.90 ± 0.09 ^bc^	3.86
SH55	91.90 ± 2.35 ^e^	2.23 ± 0.03 ^f^	4.62	1.83 ± 0.03 ^bc^	3.79	1.86 ± 0.07 ^bc^	3.84

Values are expressed as mean ± standard deviation of triplicates. Na-SG, sodium glycocholate (6 mM); Na-TA, sodium taurocholate (6 mM); bile salt mixture (6 mM; glycocholic acid, glycochenodeoxycholic acid, taurocholic acid, taurochenodeoxycholic acid, taurodeoxycholic acid); SA, specific activity (U/mg). ^a–f^ Means in same column with different lowercase letters differed significantly (*p* < 0.05). SA, specific activities (U/mg).

**Table 3:** Autoaggregation (%) and hydrophobicity (%) of 12 potential probiotic yeast isolates.

Isolate	Autoaggregation (%)			Hydrophobicity (%)		
	3 h	6 h	24 h	n-Hexane	Octane	Xylene
G1	42.3 ± 0.28 ^b^	56.7 ± 1.13 ^b^	69.8 ± 1.57 ^b^	36.8 ± 3.04 ^bcde^	42.31 ± 1.85 ^fg^	6.51 ± 2.21 ^a^
O12	58.9 ± 0.55 ^cd^	73.6 ± 0.60 ^c^	80.7 ± 0.32 ^c^	32.6 ± 5.71 ^abcd^	36.7 ± 5.24 ^cde^	25.16 ± 2.55 ^bcde^
O13	60.7 ± 0.44 ^de^	75.8 ± 1.14 ^c^	83.2 ± 0.75 ^de^	30.1 ± 1.15 ^ab^	40.65 ± 0.86 ^efg^	13.08 ± 7.56 ^ab^
O18	64.1 ± 0.51 ^fg^	78.4 ± 0.46 ^c^	82.8 ± 1.00 ^d^	31.5 ± 1.95 ^abc^	43.46 ± 3.02 ^g^	24.86 ± 4.20 ^bcde^
O21	65.1 ± 0.21 ^gh^	77.0 ± 2.41 ^c^	83.7 ± 0.13 ^de^	41.9 ± 1.45 ^de^	35.21 ± 1.07 ^bcd^	20.73 ± 2.72 ^abcd^
O26	65.6 ± 0.35 ^gh^	77.5 ± 0.75 ^c^	84.4 ± 1.11 ^def^	37.6 ± 2.76 ^bcde^	34.46 ± 1.47 ^abcd^	37.72 ± 3.31 ^e^
O36	59.2 ± 2.49 ^cd^	75.0 ± 2.64 ^c^	84.8 ± 1.01 ^ef^	42.9 ± 1.11 ^e^	42.27 ± 2.68 ^fg^	15.62 ± 2.98 ^abc^
O63	37.7 ± 0.75 ^a^	47.0 ± 2.53 ^a^	51.0 ± 0.28 ^a^	30.7 ± 2.36 ^ab^	30.67 ± 1.27 ^a^	23.71 ± 4.37 ^bcde^
O66	62.6 ± 0.34 ^ef^	77.8 ± 0.22 ^c^	82.9 ± 1.15 ^de^	24.9 ± 1.12 ^a^	31.84 ± 3.67 ^ab^	18.03 ± 1.78 ^abc^
SH40	42.8 ± 1.38 ^b^	57.2 ± 0.49 ^b^	83.7 ± 0.05 ^de^	41.2 ± 3.61 ^cde^	44.98 ± 1.57 ^g^	29.55 ± 8.17 ^cde^
SH45	66.6 ± 0.31 ^h^	75.3 ± 4.86 ^c^	86.1 ± 0.55 ^f^	33.6 ± 1.84 ^abcde^	38.51 ± 3.84 ^def^	21.19 ± 3.46 ^abcd^
SH55	58.5 ± 0.06 ^c^	71.3 ± 0.51 ^c^	80.3 ± 1.43 ^c^	28.3 ± 1.72 ^ab^	32.85 ± 1.14 ^abc^	33.11 ± 9.87 ^de^

Values are expressed as mean ± standard deviation of triplicates. ^a–h^ Means in same column with different lowercase letters differed significantly (*p* < 0.05).

**Table 4:** Coaggregation (%) and antimicrobial activity of 12 potential probiotic yeast isolates against 4 foodborne pathogens.

Isolate	*S. Typhimurium*				*E. coli* O157:H7				*S. aureus*				*L. monocytogenes*			
	3 h	6 h	24 h	A.M	3 h	6 h	24 h	A.M	3 h	6 h	24 h	A.M	3 h	6 h	24 h	A.M
G1	12.2 ± 1.53 ^b^	23.9 ± 0.46 ^a^	42.7 ± 1.79 ^a^	+++	12.8 ± 0.55 ^h^	16.5 ± 0.97 ^a^	38.3 ± 0.6 2 ^a^	+++	18.0 ± 0.82 ^f^	26.8 ± 0.97 ^f^	48.3 ± 0.98 ^e^	+	23.8 ± 0.65 ^a^	33.7 ± 0.12 ^a^	52.1 ± 0.20 ^a^	+++
O12	17.3 ± 0.01 ^c^	46.7 ± 0.54 ^cd^	59.7 ± 1.18 ^cd^	+++	46.1 ± 1.04 ^a^	51.3 ± 0.07 ^f^	64.2 ± 0.08 ^ij^	+++	23.0 ± 0.62 ^d^	48.7 ± 0.33 ^a^	62.4 ± 1.76 ^a^	+++	38.5 ± 0.45 ^d^	45.9 ± 1.15 ^b^	61.9 ± 0.83 ^c^	+++
O13	25.8 ± 0.61 ^e^	52.9 ± 1.33 ^ef^	65.3 ± 0.15 ^e^	+	38.9 ± 0.26 ^cd^	46.2 ± 0.09 ^def^	62.0 ± 0.85 ^gh^	+++	26.8 ± 0.78 ^c^	37.4 ± 0.46 ^cd^	53.8 ± 0.46 ^cd^	+	28.9 ± 0.21 ^b^	40.1 ± 0.59 ^ab^	57.2 ± 0.14 ^b^	+++
O18	35.3 ± 0.93 ^g^	58.6 ± 1.55 ^g^	65.4 ± 2.67 ^e^	+++	37.2 ± 1.04 ^d^	47.4 ± 2.45 ^def^	59.9 ± 0.86 ^ef^	+++	21.9 ± 0.08 ^de^	31.9 ± 1.16 ^e^	48.3 ± 1.06 ^e^	+++	49.9 ± 1.08 ^f^	60.2 ± 0.95 ^d^	68.9 ± 2.07 ^d^	+++
O21	21.5 ± 0.37 ^d^	50.1 ± 0.42 ^de^	62.2 ± 0.59 ^de^	+++	43.5 ± 1.05 ^ab^	51.7 ± 0.83 ^f^	65.4 ± 0.45 ^j^	+++	19.5 ± 0.78 ^ef^	37.6 ± 0.10 ^cd^	49.8 ± 1.50 ^de^	+++	47.1 ± 0.26 ^e^	57.6 ± 2.25 ^cd^	69.6 ± 1.19 ^d^	+++
O26	21.9 ± 0.84 ^d^	50.8 ± 1.08 ^de^	62.6 ± 1.03 ^de^	+++	41.1 ± 0.73 ^bc^	50.3 ± 0.38 ^f^	63.1 ± 0.11 ^hi^	+++	21.0 ± 0.70 ^def^	46.8 ± 1.94 ^ab^	60.1 ± 0.91 ^ab^	+	45.7 ± 1.09 ^e^	52.9 ± 0.30 ^c^	66.8 ± 0.62 ^d^	+++
O36	12.0 ± 0.95 ^b^	43.4 ± 1.25 ^bc^	57.4 ± 0.06 ^bcd^	+++	22.6 ± 0.54 ^f^	35.0 ± 0.42 ^c^	52.1 ± 1.21 ^c^	+++	14.1 ± 0.12 ^g^	27.3 ± 0.49 ^f^	48.5 ± 1.28 ^e^	+++	48.0 ± 0.10 ^ef^	55.2 ± 3.36 ^cd^	68.5 ± 1.12 ^d^	+++
O63	36.0 ± 2.60 ^gh^	42.6 ± 0.16 ^bc^	54.2 ± 0.36 ^b^	+++	17.2 ± 1.15 ^g^	26.9 ± 1.09 ^b^	44.4 ± 0.72 ^b^	+++	32.7 ± 0.68 ^b^	37.6 ± 1.66 ^cd^	52.7 ± 0.86 ^cde^	+++	33.0 ± 0.45 ^c^	40.0 ± 0.93 ^ab^	53.4 ± 3.19 ^ab^	+++
O66	31.6 ± 0.63 ^f^	56.0 ± 0.39 ^fg^	65.0 ± 1.23 ^e^	+++	32.4 ± 0.88 ^e^	42.9 ± 3.03 ^de^	58.5 ± 1.68 ^e^	+++	40.6 ± 1.41 ^a^	48.0 ± 0.52 ^a^	62.1 ± 0.82 ^a^	+++	52.5 ± 0.30 ^g^	60.1 ± 2.06 ^d^	70.0 ± 0.42 ^d^	+++
SH40	37.9 ± 0.00 ^h^	55.8 ± 1.41 ^fg^	67.3 ± 2.02 ^e^	+++	33.5 ± 0.24 ^e^	41.9 ± 1.92 ^d^	54.6 ± 1.45 ^d^	+++	23.1 ± 0.56 ^d^	35.3 ± 0.68 ^de^	33.3 ± 1.00 ^f^	+++	55.5 ± 0.71 ^h^	59.7 ± 2.94 ^d^	69.3 ± 2.19 ^d^	+++
SH45	9.30 ± 0.31 ^a^	40.2 ± 0.04 ^b^	55.4 ± 1.49 ^bc^	+++	30.4 ± 0.49 ^e^	44.3 ± 1.41 ^de^	60.4 ± 0.97 ^fg^	+++	34.0 ± 1.06 ^b^	43.2 ± 0.23 ^b^	58.6 ± 1.71 ^ab^	+++	47.0 ± 0.20 ^e^	59.2 ± 1.07 ^cd^	69.8 ± 2.24 ^d^	+++
SH55	18.8 ± 0.36 ^c^	48.1 ± 1.93 ^d^	58.3 ± 0.04 ^bcd^	+++	39.4 ± 0.80 ^cd^	47.9 ± 0.66 ^ef^	61.6 ± 0.07 ^fgh^	+++	34.6 ± 1.61 ^b^	39.2 ± 1.07 ^c^	56.4 ± 0.39 ^bc^	+++	29.0 ± 0.97 ^b^	37.5 ± 0.42 ^a^	57.5 ± 4.19 ^b^	+++

Values are expressed as mean ± standard error of triplicates. A.M: antimicrobial activity. ^a–j^ Means in same column with different lowercase letters differed significantly (*p* < 0.05). (+) inhibition zone 0.1 to 1.0 mm; (+++) inhibition zone > 2.1 mm.

**Table 5:** Antibiotic resistance to 6 different antibiotics and attachment to HT-29 cells.

Isolate	Antibiotic Resistance						Attachment to HT-29 Cells
	CLI	AMP	SXT	PEN	VAN	ERY	Log_10_ CFU
G1	MS	MS	MS	R	MS	S	6.66 ± 0.06 ^e^
O12	MS	S	MS	MS	R	S	6.82 ± 0.17 ^e^
O13	MS	MS	MS	R	R	MS	6.65 ± 0.06 ^e^
O18	S	S	S	S	S	S	6.27 ± 0.06 ^bcd^
O21	MS	MS	MS	S	MS	MS	6.00 ± 0.06 ^a^
O26	S	R	R	MS	S	S	6.23 ± 0.26 ^bcd^
O36	MS	MS	MS	MS	MS	MS	6.15 ± 0.04 ^abc^
O63	S	MS	MS	MS	S	S	6.16 ± 0.19 ^abc^
O66	MS	MS	MS	MS	MS	MS	6.37 ± 0.04 ^cd^
SH40	MS	MS	S	S	MS	MS	6.36 ± 0.17 ^cd^
SH45	MS	S	S	S	S	MS	6.41 ± 0.02 ^d^
SH55	MS	S	S	MS	S	S	6.06 ± 0.03 ^ab^

Values are expressed as mean ± standard deviation of triplicates. ^a^ CLI, clindamycin (2 μg); AMP, ampicillin (10 μg); SXT, trimethoprim-sulfamethoxazole (25 μg); PEN, penicillin (10 μg); VAN, vancomycin (30 μg); ERY, erythromycin (15 μg); R, resistant; MS, moderately susceptible; S, susceptible. ^a–e^ Means in same column with different lowercase letters differed significantly (*p* < 0.05).

**Table 6:** Exopolysaccharide (EPS) production and heat resistance (Log_10_ CFU/mL) of 12 potential probiotic yeast isolates.

Isolate	EPS Production	Heat Resistance (Log_10_ CFU/mL)	
		Before	After
G1	+	6.6 ± 0.01 ^a^	4.4 ± 0.02 ^a^
O12	+	7.5 ± 0.13 ^efg^	5.2 ± 0.17 ^c^
O13	+	7.7 ± 0.03 ^g^	5.3 ± 0.00 ^cd^
O18	+	7.3 ± 0.05 ^bcd^	5.6 ± 0.06 ^f^
O21	+	7.3 ± 0.02 ^bcd^	5.5 ± 0.02 ^ef^
O26	+	7.3 ± 0.07 ^bcd^	5.3 ± 0.07 ^cd^
O36	+	7.5 ± 0.00 ^def^	5.4 ± 0.03 ^cde^
O63	+	7.2 ± 0.06 ^bcd^	5.3 ± 0.17 ^cde^
O66	+	7.3 ± 0.04 ^cde^	4.7 ± 0.10 ^b^
SH40	+	7.1 ± 0.02 ^b^	5.4 ± 0.02 ^def^
SH45	+	7.6 ± 0.07 ^fg^	5.3 ± 0.13 ^cd^
SH55	+	7.2 ± 0.03 ^bc^	4.6 ± 0.24 ^ab^

Values are expressed as mean ± standard deviation of triplicates. ^a–g^ Means in same column with different lowercase letters differed significantly (*p* < 0.05). “+” denoted to ability to produce EPS.

**Table 7:** Identification of yeast isolates using ITS/5.8S ribosomal DNA and their accession numbers obtained from GenBank.

Isolate	Microorganism	Accession No	Source
G1	*Candida* sp.	OK441052	Gamed (traditional fermented dairy product)
O12	*Pichia kudriavzevii*	OK441055	Jordanian Olive
O13	*Pichia kudriavzevii*	OK441056	Jordanian Olive
O18	*Pichia kudriavzevii*	OK441057	Jordanian Olive in oil
O21	*Pichia kudriavzevii*	OK441060	Jordanian Olive in oil
O26	*Pichia kudriavzevii*	OK441064	Moroccan green olives
O36	*Pichia kudriavzevii*	OK441067	Jordanian green olives
O63	*Pichia* sp.	OK441068	Jordanian green olives
O66	*Saccharomyces cerevisiae*	OK441070	Jordanian green olives
SH40	*Pichia kudriavzevii*	OK441071	Shanklish (traditional fermented dairy product)
SH45	*Pichia kudriavzevii*	OK441072	Shanklish (traditional fermented dairy product)
SH55	*Pichia kudriavzevii*	OK441073	Shanklish (traditional fermented dairy product)

## Data Availability

Not applicable.

## Supplementary Materials

The following supporting information can be downloaded at: https://www.mdpi.com/article/10.3390/jof8050544/s1, Table S1: Acid tolerance at pH 2.5 during 24 h of incubation at 37 °C for 105 potential probiotic yeast isolates.
