# Supplementary material for: In Vitro Characterization and Identification of Potential Probiotic Yeasts Isolated from Fermented Dairy and Non-Dairy Food Products
Source: J Fungi (Basel). 2022 May 23;8(5):544. doi: 10.3390/jof8050544 (PMC9147075; doi:10.3390/jof8050544)
Supplement: Supplementary file 1 [file jof-08-00544-s001.zip › jof-1717668-supplementary.pdf]

Supplementary Materials

Table S1: Acid tolerance at pH 2.5 during 24 h of incubation at 37℃ for 105 potential probiotic yeast isolates

Isolate	Source	Survival rate (%)
G1	Gamed (dairy product)	76.1±3.8
G2	Gamed (dairy product)	74.1±3.7
G3	Gamed (dairy product)	74.9±3.7
G4	Gamed (dairy product)	69.4±3.4
G5	Gamed (dairy product)	57.4±2.8
G6	Gamed (dairy product)	85.1±4.2
G7	Gamed (dairy product)	75.1±3.7
G8	Gamed (dairy product)	111.2±5.5
G9	Gamed (dairy product)	79.1±3.9
G10	Gamed (dairy product)	83.1±4.1
O11	Egyptian Olive (fermented black olive pickle)	45.3±2.2
O12	Jardanian Olive (fermented black olive pickle)	105.8±5.2
O13	Jardanian Olive (fermented black olive pickle)	84.8±4.2
O14	Jardanian Olive (fermented black olive pickle)	70.4±3.5
O15	Jardanian Olive (fermented black olive pickle)	80.1±4.1
O16	Jardanian Olive (fermented black olive pickle)	76.1±3.8
O17	Jardanian Olive (fermented black olive pickle)	78.1±3.9
O18	Jardanian Olive in oil (fermented black olive pickle)	95.3±4.7
O19	Jardanian Olive in oil (fermented black olive pickle)	100.1±5
O20	Jardanian Olive in oil (fermented black olive pickle)	94.2±4.7
O21	Jardanian Olive in oil (fermented black olive pickle)	100.6±5
O22	Jardanian Olive in oil (fermented black olive pickle)	92.1±4.6
O23	Jardanian Olive in oil (fermented black olive pickle)	86.7±4.3
O24	Jardanian Olive in oil (fermented black olive pickle)	90.3±4.5
O25	Moroccan green olives (fermented black olive pickle)	83.8±4.1
O26	Moroccan green olives (fermented green olive pickle)	86.1±4.3
O27	Moroccan green olives (fermented green olive pickle)	100.1±5
O28	Jordanian green olives (fermented green olive pickle)	102.6±5.1
O29	Jordanian green olives (fermented green olive pickle)	119.3±5.9
O30	Jordanian green olives (fermented green olive pickle)	83.8±4.1
O31	Jordanian green olives (fermented green olive pickle)	70.3±3.5
O32	Jordanian green olives (fermented green olive pickle)	95.1±4.7
O33	Jordanian green olives (fermented green olive pickle)	106.5±5.3
O34	Jordanian green olives (fermented green olive pickle)	82.6±4.1
O35	Jordanian green olives (fermented green olive pickle)	105.1±5.2
O36	Jordanian green olives (fermented green olive pickle)	85.1±4.2
CH37	Brie cheese (soft cow's-milk cheese)	85.7±4.2

SH38	Shanklish (dried Labanah traditional fermented by fungi)	57.1±2.8
SH39	Shanklish (dried Labanah traditional fermented by fungi)	82.9±4.1
SH40	Shanklish (dried Labanah traditional fermented by fungi)	94.8±4.7
SH41	Shanklish (dried Labanah traditional fermented by fungi)	68.1±3.4
SH42	Shanklish (dried Labanah traditional fermented by fungi)	85.1±4.2
SH43	Shanklish (dried Labanah traditional fermented by fungi)	47.8±2.3
SH44	Shanklish (dried Labanah traditional fermented by fungi)	20.9±1.0
SH45	Shanklish (dried Labanah traditional fermented by fungi)	96.1±4.8
SH46	Shanklish (dried Labanah traditional fermented by fungi)	92.2±4.6
SH47	Shanklish (dried Labanah traditional fermented by fungi)	80.9±4.0
SH48	Shanklish (dried Labanah traditional fermented by fungi)	68.8±3.4
SH49	Shanklish (dried Labanah traditional fermented by fungi)	86.5±4.3
SH50	Shanklish (dried Labanah traditional fermented by fungi)	87.1±4.3
SH51	Shanklish (dried Labanah traditional fermented by fungi)	75.1±3.7
SH52	Shanklish (dried Labanah traditional fermented by fungi)	71.4±3.5
SH53	Shanklish (dried Labanah traditional fermented by fungi)	73.3±3.6
SH54	Shanklish (dried Labanah traditional fermented by fungi)	133.5±6.6
SH55	Shanklish (dried Labanah traditional fermented by fungi)	86.9±4.3
SH56	Shanklish (dried Labanah traditional fermented by fungi)	63.9±3.1
D57	Date fruit	44.6±2.2
D58	Date fruit	34.3±1.7
D59	Date fruit	50.2±2.5
O60	Egyptian Olive (fermented black olive pickle)	87.1±4.3
O61	Egyptian Olive (fermented black olive pickle)	96.4±4.8
O62	Jordan green olives (fermented black olive pickle)	81.6±4.0
O63	Jordanian green olives (fermented black olive pickle)	115.5±5.7
O64	Jordanian green olives (fermented black olive pickle)	96.7±4.8
O65	Jordanian green olives (fermented black olive pickle)	92.1±4.6
O66	Jordanian green olives (fermented black olive pickle)	91.7±4.5
O67	Jordanian green olives (fermented black olive pickle)	40.2±2.0
O68	Jordanian green olives (fermented black olive pickle)	44.9±2.2
O69	Gamed (dairy product)	75.4±3.7
G70	Gamed (dairy product)	59.6±2.9
G71	Gamed (dairy product)	79.2±3.9
G72	Gamed (dairy product)	51.1±2.5
G73	Gamed (dairy product)	70.8±3.5
G74	Gamed (dairy product)	37.5±1.8
G75	Gamed (dairy product)	79.2±3.9
G76	Gamed (dairy product)	19.4±0.9
G77	Gamed (dairy product)	88.6±4.4
G78	Gamed (dairy product)	78.3±3.9
G79	Gamed (dairy product)	51.5±2.5
G80	Gamed (dairy product)	75.7±3.7

G81	Gamed (dairy product)	50.2±2.5
G82	Gamed (dairy product)	74.1±3.7
G83	Gamed (dairy product)	36.1±1.8
G84	Gamed (dairy product)	77.2±3.8
G85	Gamed (dairy product)	101.8±5
G86	Gamed (dairy product)	70.4±3.5
G87	Gamed (dairy product)	93.5±4.6
G88	Gamed (dairy product)	67.9±3.3
G89	Gamed (dairy product)	70.2±3.5
G90	Gamed (dairy product)	64.8±3.2
G91	Gamed (dairy product)	67.8±3.3
G92	Gamed (dairy product)	87.8±4.3
G93	Gamed (dairy product)	53.1±2.6
G94	Gamed (dairy product)	70.2±3.5
G95	Gamed (dairy product)	69.2±3.4
SH96	Shanklish (dried Labanah traditional fermented by fungi)	85.7±4.2
SH97	Shanklish (dried Labanah traditional fermented by fungi)	89.4±4.4
SH98	Shanklish (dried Labanah traditional fermented by fungi)	92.5±4.6
SH99	Shanklish (dried Labanah traditional fermented by fungi)	90.8±4.5
SH100	Shanklish (dried Labanah traditional fermented by fungi)	89.9±4.4
SH101	Shanklish (dried Labanah traditional fermented by fungi)	75.1±3.7
SH102	Shanklish (dried Labanah traditional fermented by fungi)	79.1±3.9
SH103	Shanklish (dried Labanah traditional fermented by fungi)	83.6±4.1
SH104	Shanklish (dried Labanah traditional fermented by fungi)	85.8±4.2
SH105	Shanklish (dried Labanah traditional fermented by fungi)	86.6±4.3
